# Divergent environmental constraints shape the spatial patterns of annual gross primary productivity in China’s terrestrial ecosystems

**DOI:** 10.3389/fpls.2026.1829908

**Published:** 2026-06-11

**Authors:** Sha Lei, Ping Zhou, Hui Chen, Ping Yan, Jiaying Lin, Zhaowei Tan, Junxiang Huang, Jiahui Xue

**Affiliations:** 1 Guangzhou Institute of Geochemistry, Chinese Academy of Sciences, Guangzhou, China; 2 Guangzhou Institute of Geography, Guangdong Academy of Sciences, Guangzhou, China; 3 Guangdong Nanling Forest Ecosystem National Field Scientific Observation and Research Station, Guangzhou Institute of Geography, Guangdong Academy of Sciences, Guangzhou, China; 4 University of Chinese Academy of Sciences, Beijing, China; 5 National Ecological Science Data Center Guangdong Branch, Guangzhou, China; 6 School of Geography and Remote Sensing, Guangzhou University, Guangzhou, China

**Keywords:** annual gross primary productivity (AGPP), ecosystem types, eddy covariance (EC), machine learning, structure equation modeling (SEM)

## Abstract

**Introduction:**

Annual Gross Primary Productivity (AGPP) is a key indicator of terrestrial ecosystem carbon sequestration. However, its spatial variability and biome-specific controls remain poorly understood.

**Methods:**

Here, we analyzed AGPP across forests, grasslands, croplands, and wetlands in China using data from 166 eddy covariance flux sites. We applied SHapley Additive exPlanations (SHAP) and structural equation modeling (SEM) to quantify the relative importance of environmental variables and to disentangle their direct and indirect effects on AGPP.

**Results:**

Our results revealed clear productivity patterns: forests exhibited the highest AGPP, followed by wetlands, croplands, and grasslands. In forests, AGPP was positively associated with temperature and precipitation but negatively related to high vapor pressure deficit (VPD). In grasslands, precipitation enhanced AGPP both directly and indirectly via vegetation structure (leaf area index), whereas excessive radiation constrained productivity. High soil organic carbon (SOC) was associated with lower AGPP in wetlands and croplands, likely reflecting nutrient immobilization under waterlogged conditions and the impacts of intensive agricultural management.

**Discussion:**

These findings highlight biome-specific responses of AGPP to climatic conditions, soil properties, and vegetation structure, and provide insights for improving carbon cycle models and guiding targeted ecosystem management.

## 1 Introduction

Annual gross primary productivity (AGPP) is a critical metric in ecosystem ecology, representing the total amount of carbon dioxide sequestered from the atmosphere through photosynthesis over one year (Anav et al., 2015; Zhu et al., 2022). As a fundamental indicator of ecosystem function, AGPP is a key determinant of the capacity of terrestrial ecosystems to act as carbon sinks (Piao et al., 2009; Zhu et al., 2016). The variation in AGPP not only reflect the long-term direction and magnitude of carbon assimilation but also directly influence the dynamics of the terrestrial carbon cycle (Zhang et al., 2020; Wang et al., 2020). Understanding AGPP variation and their driving factors is essential for assessing spatiotemporal variations in carbon sequestration, which is vital for ecosystem management, climate change mitigation, and the development of carbon neutrality strategies (Piao et al., 2022; Gui et al., 2025).

Extensive research utilizing data from eddy covariance networks has examined the spatial variability of AGPP across diverse regions and ecosystem types (Zhu et al., 2023; Yu et al., 2024). Substantial progress has been made in elucidating the dominant influence of mean annual air temperature (MAT) and mean annual precipitation (MAP) on the spatial patterns of AGPP (Guo et al., 2012; Yu et al., 2013; Zhu et al., 2022). Beyond these climatic drivers, recent studies have highlighted the synergistic contributions of vegetation phenology and physiological processes to observed AGPP patterns (Zhou et al., 2016; Xu et al., 2019; Gui et al., 2024). Constrained by the scarcity of observational data, earlier investigations have generally bifurcated into either broad regional analyses—spanning China (Yu et al., 2013; Han et al., 2020), Asia (Piao et al., 2012; Chen et al., 2013), the Northern Hemisphere (Zhuang et al., 2011; Zhi et al., 2014), and the globe (Levy et al., 2004; Pan et al., 2011)—or studies focused on specific ecosystem types, such as forests (Xu et al., 2014; Zhou et al., 2021), grasslands (Wang et al., 2021; Yang et al., 2022), and wetlands (Wang et al., 2023). Critically, these studies often employ fixed model parameters or data-driven approaches that tend to overlook the indirect effects of ecosystem responses on AGPP variation. Such ecosystem responses, encompassing structural attributes (e.g., leaf area index, LAI) and functional characteristics (e.g., productivity per unit leaf area, PAGPP), are dynamically shaped by environmental conditions and play a key role in modulating AGPP (Gower et al., 1999; Zhang et al., 2022; Han et al., 2025).

China’s terrestrial landscape is characterized by vast environmental gradients, ranging from humid tropical forests to arid deserts and high-altitude alpine meadows. Within this heterogeneous context, different ecosystem types exhibit distinct sensitivities and adaptive responses to environmental changes, resulting in significant spatial heterogeneity in AGPP (Xie et al., 2021; Zhou et al., 2022). Specifically, forests, grasslands, croplands, and wetlands display divergent productivity patterns driven by contrasting biophysical constraints (Xu et al., 2021; Liu et al., 2024; Yuan et al., 2025). For instance, forest productivity is often primarily regulated by radiation and thermal conditions, supported by their complex canopy structure and high leaf area index (LAI) (Li et al., 2023). In contrast, the dynamics of grasslands and wetlands are typically more tightly coupled with soil moisture availability and precipitation variability, reflecting a strong dependence on hydrological conditions (Huang et al., 2024). Such divergence underscores the critical necessity of incorporating ecosystem-specific characteristics into carbon dynamic modeling. Neglecting these spatial differences and the unique acclimation strategies of each biome can lead to substantial biases. Consequently, models may fail to accurately capture the interannual variations and spatial discrepancies in measured AGPP, thereby undermining the reliability of regional carbon cycle assessments and the effectiveness of ecosystem management strategies.

Since 2002, the Chinese Terrestrial Ecosystem Flux Research Network (ChinaFLUX) has conducted extensive *in situ* flux measurements across a wide range of ecosystems, providing a robust data foundation for exploring the spatial variability of AGPP in China’s terrestrial ecosystems (Yu et al., 2006, 2016, 2024). The rapid advancement of eddy covariance techniques has significantly increased the availability and quality of flux observations, thereby enabling more detailed investigations into how different ecosystem types contribute to AGPP spatial variability and enhancing our understanding of AGPP distribution patterns across China. To address these limitations, this study aims to utilize *in situ* observations from ChinaFLUX, in combination with machine learning and structural equation modeling (SEM), to analyze the spatial distribution patterns of vegetation productivity (AGPP) across different ecosystem types and to identify the key environmental drivers underlying these patterns. This study focuses primarily on ecosystem-specific spatial variation in AGPP across China, using multi-year site observations to improve sample coverage rather than to quantify national-scale temporal variation. The primary objectives of this research are to: (1) delineate the spatial patterns of AGPP across diverse ecosystem types in China and assess their regional contributions; (2) evaluate the relative roles of environmental factors and ecosystem responses in explaining AGPP variation; and (3) elucidate the pathway patterns through which these responses are associated with AGPP differences across ecosystems. This study builds on existing research by addressing gaps in understanding the spatial distribution of AGPP and its environmental drivers across China’s diverse ecosystems. By focusing on the interactions between environmental factors and ecosystem responses, we aim to provide novel insights into the mechanisms underlying AGPP variations. Our findings will not only enhance the scientific understanding of carbon cycling in heterogeneous landscapes but also offer practical guidance for optimizing ecosystem management and developing effective climate adaptation strategies.

## 2 Materials and methods

### 2.1 Site data and processing

**Figure 1:**
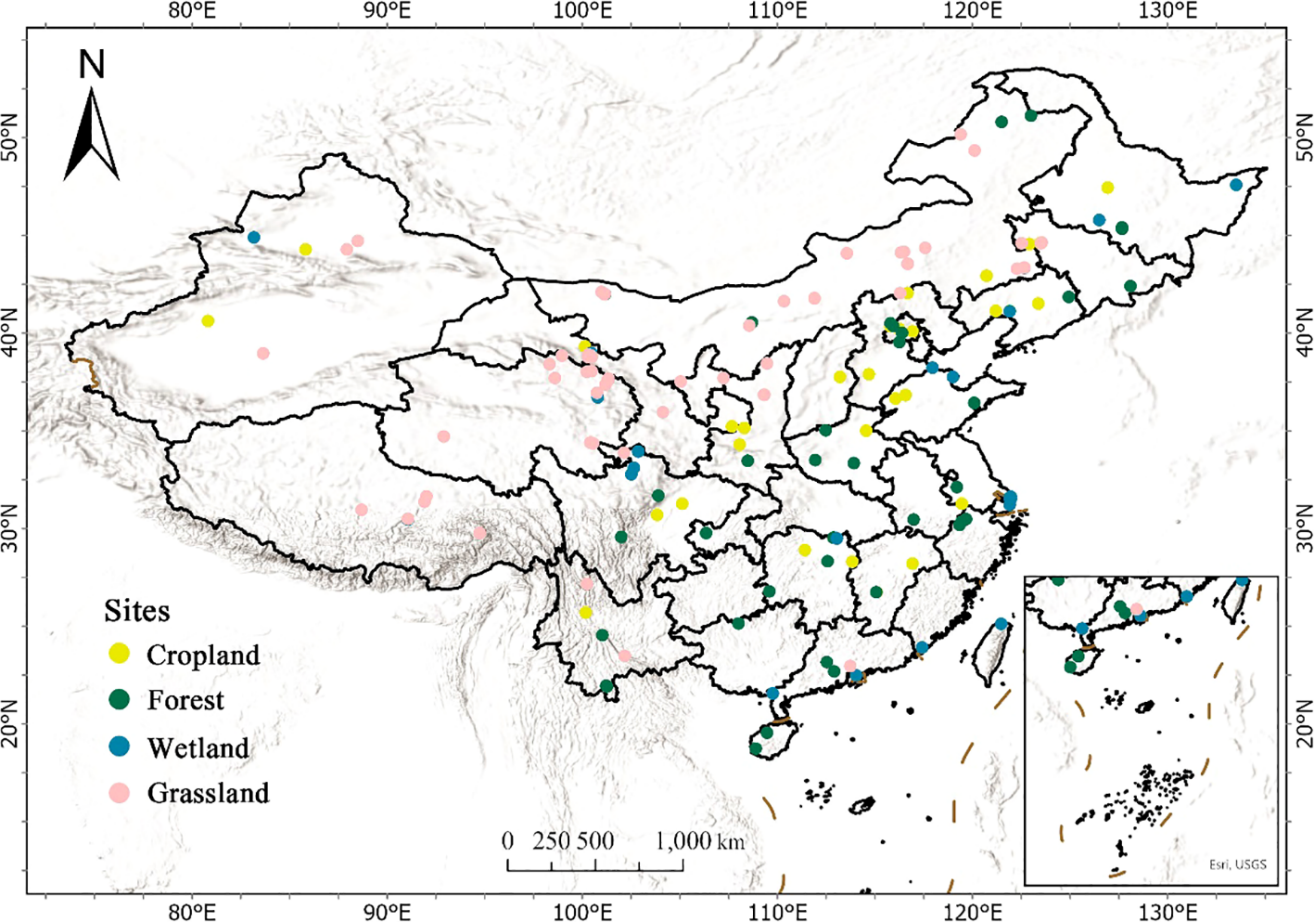
Spatial distribution of the 166 eddy covariance flux sites across China by ecosystem type.

The site data used in this study included both direct AGPP measurements and auxiliary site-level variables for upscaling AGPP to the regional scale. Specifically, we utilized a publicly available dataset of annual gross primary productivity (AGPP) for China’s terrestrial ecosystems from 2000 to 2020 (Zhu et al., 2023). This dataset comprises 641 site-year records from 166 flux observation sites (**Figure 1**), including 43 forest sites, 59 grassland sites, 38 cropland sites, and 26 wetland sites (https://www.scidb.cn/detail?dataSetId=b496b208f51e44fcaf326e8b0f792c34&version=V3). Among these, 24 sites have observation periods exceeding seven years, providing sufficient temporal coverage for assessing spatial variation in AGPP. The auxiliary site-level data were categorized into three major groups: climatic variables, soil properties (e.g., soil organic carbon, SOC), and biotic structural variables (Zhu et al., 2023). Climatic variables included mean annual temperature (MAT), mean annual precipitation (MAP), annual photosynthetically active radiation (PAR), annual potential evapotranspiration (PET), mean annual vapor pressure deficit (VPD), and mean annual atmospheric CO_2_ concentration (CO_2_). Soil properties comprised mean annual soil moisture (SM), soil organic carbon content (SOC), and total soil nitrogen content (STN), which together represent soil water status, nutrient availability, and long-term soil conditions. Biotic structural variables included mean leaf area index (LAI) and maximum leaf area index (MLAI), which characterize vegetation structure and act as intermediate response variables linking environmental conditions to AGPP. These variables were not treated equivalently as external drivers, but rather represent different components of the ecosystem, including climatic forcing, soil conditions, and vegetation structure. All auxiliary variables were extracted from gridded datasets with a spatial resolution of approximately 1 km. Although the dataset spans multiple terrestrial ecosystems across China, the spatial distribution of flux sites is not uniform across all regions, and only a subset of sites has relatively long observation records. Therefore, the dataset is more suitable for evaluating ecosystem-specific spatial variation based on available site-year observations than for representing all regions with equal statistical coverage.

Regional auxiliary datasets covering climate, soil, and biological attributes were mainly derived via downscaling from publicly available sources, with a spatial resolution of approximately 1 km over the period 2000–2020 (Zhu et al., 2022)(**Table 1**). MAT, MAP, and VPD were derived from 0.5° time-series data provided by the Climate Research Unit (CRU) (Harris et al., 2020) and subsequently downscaled using a delta method (Peng et al., 2019), incorporating long-term climatology from the WorldClim dataset (www.worldclim.org). PAR was sourced from the Global Land Surface Satellite (GLASS) PAR product (version V60) (Cheng et al., 2015), with a native spatial resolution of 0.05° and a daily temporal resolution. Annual PAR values were calculated by aggregating daily data and then downscaled using spline interpolation. PET, obtained from monthly CRU time series (0.5°) (Harris et al., 2020), was processed similarly: annual values were computed by summing monthly data and downscaled via spline interpolation. In this study, PET was used as an indicator of atmospheric evaporative demand rather than as a measure of actual evapotranspiration. Atmospheric CO_2_ concentration was estimated using the ideal gas law, incorporating CO_2_ molar mass (44 g mol^−1^), concentration data from Mauna Loa, local air pressure, and MAT to compute the molar volume of CO_2_ (Zhu et al., 2023). SM was derived from a fine-resolution microwave surface soil moisture (SSM) dataset (Meng et al., 2021), which has a spatial resolution of 0.05° and monthly temporal resolution. Annual SM values were obtained by averaging monthly data and then downscaled using spline interpolation. SOC was calculated based on soil organic carbon density (g C kg^−1^), bulk density (g cm^−3^), a soil depth of 5 cm, and a 1 m² reference area, using a global soil dataset with no interannual variability (Shangguan et al., 2014). STN was computed using the same method. LAI and MLAI were obtained from validated Moderate Resolution Imaging Spectroradiometer (MODIS) products, specifically the MCD15A2H and MOD15A2H LAI datasets, and land cover data from MCD12Q1 (Yuan et al., 2011). These MODIS products have a native spatial resolution of 30 arc seconds and 8-day temporal resolution. Annual LAI and MLAI values were calculated as the mean and maximum across the growing season, respectively.

**Table 1:** Information of regional auxiliary datasets used in this study.

Abbreviation	Full name/variables	Datasets/products	Spatial resolution	Time resolution	Time period	Data sources & references
MAT	Mean annual temperature	CRU TS + WorldClim	~1 km (downscaled)	Annual	2000–2020	Harris et al., 2020; Peng et al., 2019
MAP	Mean annual precipitation	CRU TS + WorldClim	~1 km (downscaled)	Annual	2000–2020	Harris et al., 2020; Peng et al., 2019
VPD	Vapor pressure deficit	CRU TS + WorldClim	~1 km (downscaled)	Annual	2000–2020	Harris et al., 2020; Peng et al., 2019
PAR	Photosynthetically active radiation	GLASS PAR (V60)	~1 km	Daily → Annual	2000–2020	Cheng et al., 2015
PET	Potential evapotranspiration	CRU TS	~1 km (downscaled)	Monthly → Annual	2000–2020	Harris et al., 2020
CO_2_	Atmospheric CO_2_ concentration	Mauna Loa CO_2_ + local climate	~1 km	Annual	2000–2020	Zhu et al., 2023
SM	Soil moisture	Microwave SSM dataset	~1 km	Monthly → Annual	2000–2020	Meng et al., 2021
SOC	Soil organic carbon	Global soil dataset	~1 km	Static (no interannual variation)	–	Shangguan et al., 2014
STN	Soil total nitrogen	Global soil dataset	~1 km	Static (no interannual variation)	–	Shangguan et al., 2014
LAI	Leaf area index	MODIS MCD15A2H	~1 km	8-day → Annual mean	2000–2020	Yuan et al., 2011
MLAI	Maximum leaf area index	MODIS MOD15A2H	~1 km	8-day → Annual max	2000–2020	Yuan et al., 2011
Land cover	Land cover type	MODIS MCD12Q1	~1 km	Annual	2000–2020	Yuan et al., 2011

After preprocessing, all auxiliary variables were resampled or downscaled to the same 1 km grid and matched to each flux site according to site coordinates. For each site-year record, the value of each gridded variable was extracted from the grid cell containing the flux tower using a nearest-neighbor sampling approach. Dynamic variables (MAT, MAP, PAR, PET, VPD, CO_2_, SM, LAI, and MLAI) were matched year by year to the corresponding AGPP observation year, whereas static soil variables (SOC and STN) were assigned to all years for a given site.

### 2.2 Statistical analysis

In this study, each site-year record was treated as an observational unit. Although the dataset includes multi-year observations from 2000 to 2020, the analytical framework was primarily designed to characterize ecosystem-specific spatial variation in AGPP across site-year observations rather than temporal trajectories within individual sites. Site‐level AGPP, climatic, soil, and biological data were assembled to conduct a comprehensive analysis of (1) AGPP differences among ecosystem types, (2) correlations between AGPP and its drivers, and (3) the biotic and abiotic factors governing AGPP spatial variability (Section 2.3).

One‐way analysis of variance (ANOVA, *α* = 0.05) was employed to test for significant differences in AGPP across ecosystem types (Liu et al., 2024).

We performed Pearson correlation analysis to assess the relationships between AGPP and its potential drivers within each ecosystem type (forest, grassland, cropland, wetland) (Isbell et al., 2015; Zhu et al., 2023). Annual AGPP and auxiliary variables (MAT, MAP, PAR, PET, VPD, CO_2_, SM, SOC, STN, LAI, MLAI) were standardized (mean = 0, SD = 1). For each ecosystem category, we calculated Pearson’s *r* for all variable pairs and tested significance with two‐tailed tests at α = 0.05, 0.01, and 0.001 (denoted *, **, ***). These analyses identified key climatic, soil, and biological controls on AGPP and revealed inter‐driver collinearity. All analyses were conducted using RStudio (Posit PBC, Boston, MA, USA).

### 2.3 Analysis of AGPP controls based on machine learning

#### 2.3.1 Selection of variables

The long-term variation of AGPP is influenced by a combination of climatic conditions, soil properties, and vegetation structure (Klumpp et al., 2011; Zhu et al., 2022). Based on previous studies, eleven potential variables were initially selected, including MAT, MAP, PAR, PET, VPD, CO_2_, SM, SOC, STN, LAI, and MLAI (Zhu et al., 2023). To reduce redundancy and avoid multicollinearity among variables, a multicollinearity test was conducted prior to the analysis (Jia et al., 2025). As a result, ten variables (MAT, MAP, PAR, PET, VPD, CO_2_, SM, SOC, STN, and LAI) were retained as the final set of driving factors for subsequent analysis. These variables represent different components of the ecosystem, including climatic forcing, soil conditions (e.g., soil organic carbon, SOC), and biotic structural attributes (e.g., leaf area index, LAI), rather than being treated uniformly as independent external drivers.

#### 2.3.2 Identification of key variables associated with AGPP

To identify the variables associated with AGPP, SHapley Additive exPlanations (SHAP) analysis was employed as a *post-hoc* interpretability method. Before applying SHAP to quantify the contribution of each factor, an XGBoost (Extreme Gradient Boosting) model was constructed to capture the relationship between the selected variables and AGPP. XGBoost is a powerful and widely used machine learning algorithm based on gradient boosting decision trees, well-suited for feature importance analysis and predictive modeling (Lundberg and Lee, 2017; Chen and Guestrin, 2016). In this study, the dataset was split into training and testing subsets (80:20) for each ecosystem type (forest, grassland, cropland, wetland), and hyperparameters were optimized via grid search with 10-fold cross-validation. Candidate hyperparameters included n_estimators, max_depth, learning_rate, subsample, colsample_bytree, and min_child_weight. The final parameter sets for each ecosystem-specific model are reported in **Table 2**. SHAP values were calculated *post hoc* to quantify the contribution of each predictor to model outputs. Variable importance was measured as the mean absolute SHAP value across all samples, and relative contributions were expressed as percentages of the total SHAP sum. Pearson correlation analysis was retained to provide a supplementary linear comparison. It should be noted that SHAP reflects model-based associations rather than causal effects. Bootstrap analyses were performed to assess robustness. For each resampled dataset, the XGBoost model was retrained and SHAP values recalculated, and the mean and 95% confidence interval of relative contributions were summarized.

**Table 2:** Optimal XGBoost hyperparameters for ecosystem-specific AGPP models.

Ecosystem	n_estimators	max_depth	learning_rate	subsample	colsample_bytree	min_child_weight
Forest	500	6	0.05	0.8	0.8	1
Grassland	400	5	0.08	0.9	0.7	1
Cropland	600	7	0.04	0.85	0.9	2
Wetland	450	6	0.06	0.8	0.8	1

SHAP, rooted in cooperative game theory, quantifies the marginal contribution of each input feature to the model’s output (Lundberg and Lee, 2017; Aksoy et al., 2024). It assigns a SHAP value to each feature, indicating its impact on the prediction for a given sample. The overall importance of each driving factor was then assessed by calculating the mean absolute SHAP value across all observations (Cha et al., 2021; Kim and Kim, 2022).


$$y=f_{0}+\sum_{i=1}^{M}f_{1}$$

where *y*, *i*, *f_0_* and *f_1_* denote the output of the explanatory model, the number of drivers, the mean value of all training data and the corresponding attribute values for each driver feature, respectively. The contribution of each driver feature *X_i_* to the model output was calculated using the following formula:


$$\emptyset \left(X_{i}\right)=\sum_{T\in N_{\left\{i\right\}}}\frac{\left|T\right|!\left(N-\left|T\right|-1\right)!}{\left|T!\right|}\left(f\left(T\cup \left\{i\right\}\right)-f\left(T\right)\right)$$

where N denotes the number of driver features, and N{*i*} denotes the all input datasets, T denotes the driver feature set in N{*i*}, f(*T*) denotes the model output of feature *T*, and f(*T* ∪ {*i*}) denotes adding feature *X_i_* to the model output of feature *T*. Corresponding model outputs are obtained for each driver data, with the SHAP value indicating the contribution of each driver feature. The higher the SHAP value, the greater the contribution of the driver feature and vice versa. Moreover, Pearson’s analysis was used to measure the correlation between drivers and AGPP.

#### 2.3.3 Structural equation modeling (SEM)

We employed piecewise structural equation modeling (SEM) framework to disentangle the direct and indirect influences of climatic, soil, and biological drivers on annual gross primary productivity (AGPP) within forest, grassland, cropland, and wetland systems. All analyses were performed in RStudio (Posit PBC, Boston, MA, USA) using the piecewiseSEM package (Lefcheck, 2016). Three linear submodels were specified and fitted via ordinary least squares: (1) leaf area index (LAI), included as a biotic structural variable reflecting vegetation response (Liu et al., 2018), as a function of mean annual temperature (MAT), mean annual precipitation (MAP), photosynthetically active radiation (PAR), vapor pressure deficit (VPD), potential evapotranspiration (PET), and atmospheric CO_2_ concentration; (2) soil moisture (SM) as a function of MAT, MAP, PAR, VPD, and PET; and (3) AGPP as a function of LAI, SM, MAT, MAP, PAR, VPD, PET, and CO_2_. All predictor variables were standardized (mean = 0, SD = 1) prior to model fitting. Submodels were then integrated into a piecewise SEM via the `psem()` function, which implements Shipley’s d-separation test (Fisher’s C statistic; Shipley, 2009) to assess global model adequacy and calculates Akaike’s Information Criterion (AIC) for model comparison. Standardized path coefficients (*β*) and associated *p*-values (*α* = 0.05) were extracted to quantify total, direct, and indirect effects on AGPP. Ecosystem-specific SEMs were conducted to compare explained variances (*R*²) and pathway strengths among the four ecosystem types (Grace et al., 2010).

SHAP and SEM were used complementarily: SHAP identifies key predictors and captures nonlinear effects, whereas SEM elucidates direct and indirect causal pathways. Both methods consistently highlighted major drivers—MAT, MAP, LAI, VPD, and SM—though differences arise from SHAP’s nonlinear perspective versus SEM’s linear/path-based framework. Uncertainty in SHAP and SEM results was assessed via bootstrap resampling and evaluation of standardized path coefficients, respectively.

## 3 Results

### 3.1 Statistical characteristics of AGPP and environmental factors in different terrestrial ecosystems

**Figure 2:**
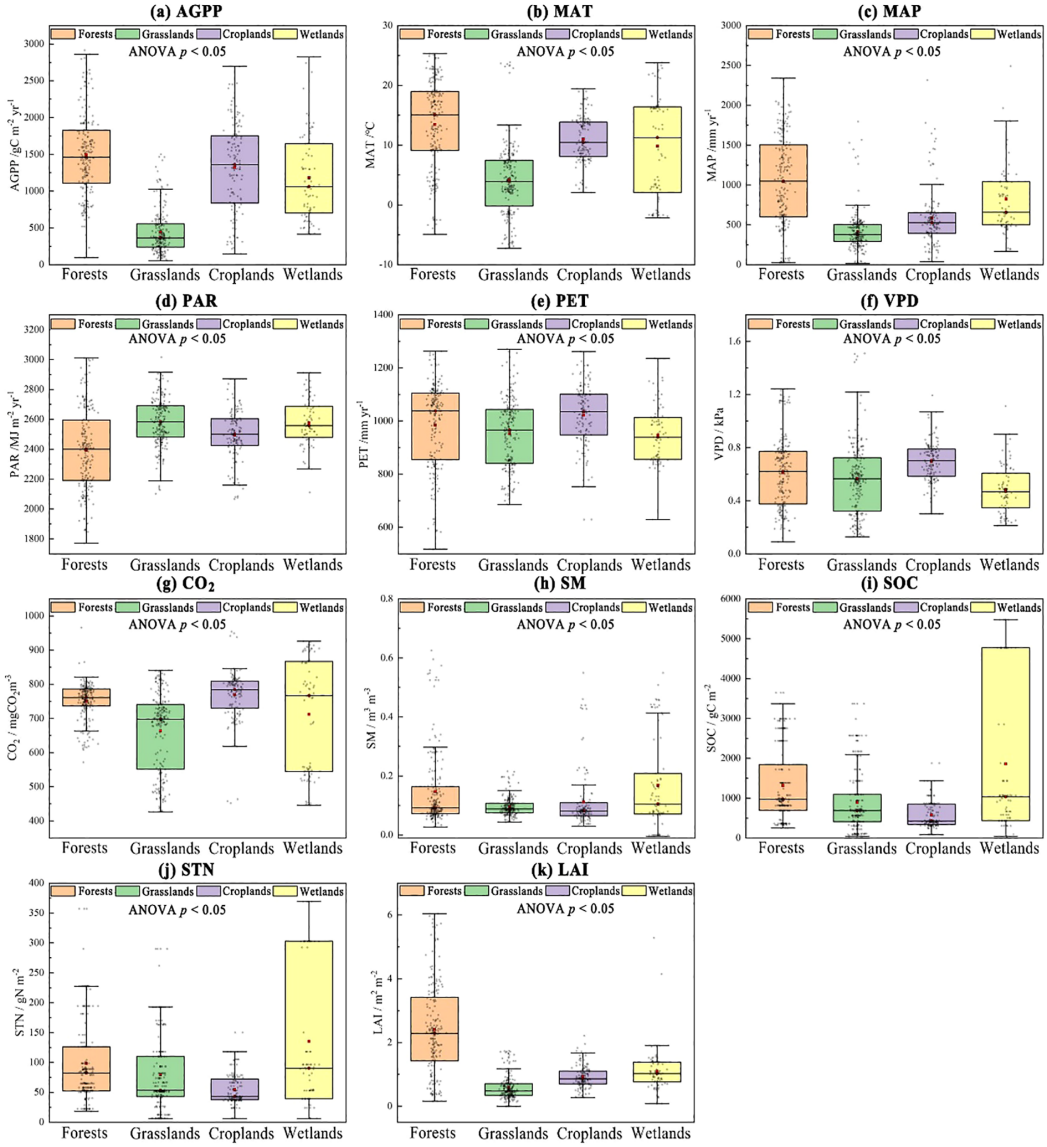
Statistics analysis of AGPP and environmental variables in different ecosystem types. **(a)**AGPP is annual gross primary productivity. **(b)** MAT is annual mean air temperature. **(c)** MAP is annual precipitation. **(d)** PAR is annual photosynthetically active radiation. **(e)** PET is annual potential evapotranspiration. **(f)** VPD is annual mean vapor pressure deficit. **(g)** CO_2_ is annual mean CO_2_ mass concentration. **(h)** SM is annual mean soil moisture. **(i)** SOC is annual mean soil organic carbon content. **(j)** STN is annual mean soil total nitrogen content. **(k)** LAI is mean leaf area index. The median is denoted by the black line, and the means are denoted by the red point. The red dashed line represents the overall mean AGPP, MAT, MAP, PAR, PET, VPD, CO_2_, SM, SOC, STN and LAI across all ecosystem types.

The statistical distributions of AGPP and key environmental factors across forests, grasslands, croplands, and wetlands from 2000 to 2020 are presented in **Figure 2**. ANOVA results indicated significant differences among ecosystem types (*p* < 0.05). AGPP exhibited substantial variation across vegetation types (**Figure 2a**). Forests recorded the highest productivity (1494.76 ± 39.16 g C m^−2^ yr^−1^), followed by wetlands (1181.21 ± 66.15 g C m^−2^ yr^−1^), while grasslands showed the lowest values (442.18 ± 20.26 g C m^−2^ yr^−1^). These disparities reflect distinct levels of ecosystem productivity, likely driven by variations in vegetation structure, biomass accumulation, and prevailing environmental conditions. Climatically, forests and wetlands were characterized by the highest mean annual temperature (MAT) and precipitation (MAP) (**Figures 2b, c**). Forests averaged 13.45 ± 0.52 °C and 1042.15 ± 40.34 mm, while wetlands averaged 9.82 ± 0.97 °C and 822.61 ± 55.11 mm, suggesting that hydrothermal conditions strongly influence AGPP. Regarding radiative and evaporative factors, forests and wetlands received slightly higher PAR (**Figure 2d**). Potential evapotranspiration (PET) was highest in croplands (1022.16 ± 10.17 mm yr^−1^) and forests (984.32 ± 11.17 mm yr^−1^), indicating potential water limitations in these ecosystems (**Figure 2e**). Furthermore, vapor pressure deficit (VPD) peaked in forests (0.61 ± 0.02 kPa) (**Figure 2f**), influencing evaporative demand and stomatal regulation. Soil properties also played a pivotal role (**Figures 2h, i**). Soil moisture (SM) and soil organic carbon (SOC) were significantly higher in wetlands (0.17 ± 0.02 m³ m^−3^ and 1859.41 ± 214.84 g C m^−2^, respectively). In contrast, grasslands (SM: 0.10 ± 0.002 m³ m^−3^; SOC: 894.28 ± 54.26 g C m^−2^) and croplands (SM: 0.11 ± 0.01 m³ m^−3^; SOC: 574.80 ± 29.35 g C m^−2^) exhibited lower values, reflecting differences in water retention and organic matter accumulation. Similarly, soil total nitrogen (STN) followed the variation of SOC, highlighting variations in nutrient availability. Biologically, Leaf Area Index (LAI) was highest in forests (2.41 ± 0.10 m² m^−2^), supporting their superior productivity. Conversely, lower LAI values in grasslands (0.59 ± 0.03 m² m^−2^) and croplands (0.93 ± 0.03 m² m^−2^) may limit their AGPP potential. Collectively, these findings emphasize the integrated roles of climate, soil properties, and vegetation structure in shaping the spatial distribution of AGPP across China’s terrestrial ecosystems.

### 3.2 Correlation between AGPP and influencing factors in different terrestrial ecosystems

**Figure 3:**
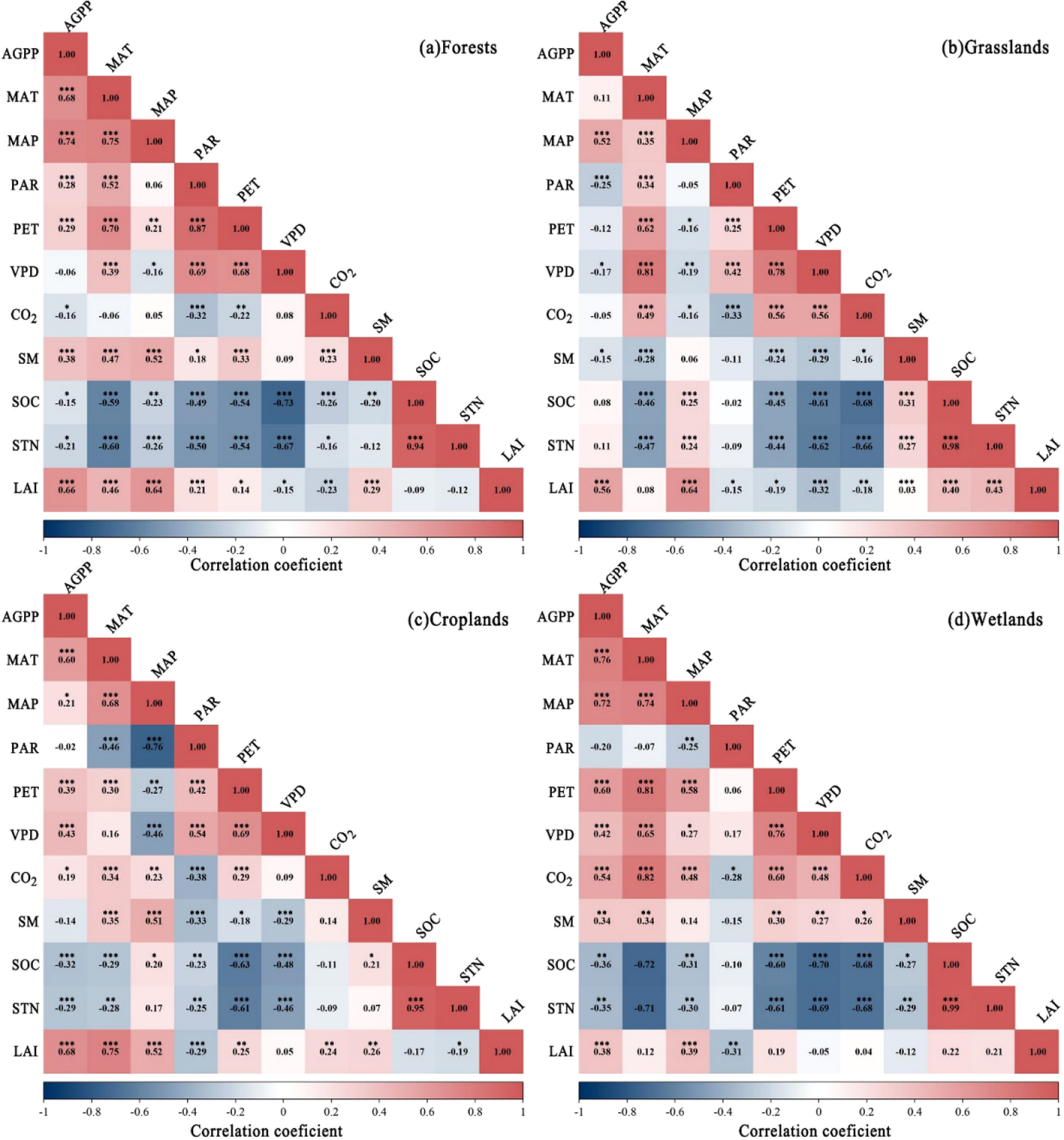
The correlation matrix heatmap between AGPP and environmental factors in different ecosystem types based on Pearson’s correlation analysis during 2000 to 2020. **(a–d)** represent correlation matrix heatmaps of forests, grasslands, croplands and wetlands, respectively. The red colors represent positive correlation coefficients, and the blue colors represent negative correlation coefficients (*: *p* < 0.05; **: *p* < 0.01; ***: *p* < 0.001).

Pearson correlation analysis was conducted to elucidate the linear relationships between AGPP and environmental drivers across four ecosystem types from 2000 to 2020 (**Figure 3**). Overall, the dominant controlling factors of AGPP varied substantially among ecosystems, exhibiting distinct dependencies on climatic, soil, and biological variables. In forests ecosystems (**Figure 3a**), AGPP exhibited robust positive correlations with precipitation and temperature, identifying MAP (*r* = 0.74, *p* < 0.001) and MAT (*r* = 0.68, *p* < 0.001) as the primary climatic drivers. Vegetation structure also played a critical role, with LAI showing a strong positive correlation (*r* = 0.66, *p* < 0.001). Notably, contrary to the weak negative associations observed in soil organic carbon (SOC, *r* = −0.15, *p* < 0.05), soil moisture (SM) demonstrated a significant positive influence (*r* = 0.38, *p* < 0.001), suggesting that moisture availability remains a limiting factor for forest productivity alongside canopy structure. In grasslands (**Figure 3b**), AGPP was strongly regulated by vegetation structure and water supply. LAI emerged as the most significant correlate (*r* = 0.56, *p* < 0.001), followed closely by MAP (*r* = 0.52, *p* < 0.001). Unlike forests, temperature (MAT) showed no significant correlation (*r* = 0.11). Interestingly, PAR exhibited a negative correlation (*r* = −0.25, *p* < 0.001), potentially indicating that high radiation periods coincide with water-stressed conditions that inhibit growth.

For croplands (**Figure 3c**), AGPP was significantly driven by biological and thermal factors. LAI (*r* = 0.68, *p* < 0.001) and MAT (*r* = 0.60, *p* < 0.001) showed the strongest positive correlations, highlighting the importance of canopy development and thermal accumulation for crop yield. Vapor pressure deficit (VPD) also exhibited a positive correlation (*r* = 0.43, *p* < 0.001), whereas SOC displayed a significant negative relationship (*r* = −0.32, *p* < 0.001), likely reflecting the impact of intensive anthropogenic management practices such as tillage and fertilization. In wetland ecosystems (**Figure 3d**), AGPP showed the highest sensitivity to climatic variables among all types. MAT (*r* = 0.76, *p* < 0.001) and MAP (*r* = 0.72, *p* < 0.001) were the dominant drivers, emphasizing a strong dependence on hydrothermal conditions. While SM showed a positive correlation (*r* = 0.34, *p* < 0.01), SOC exhibited a significant negative correlation (*r* = −0.36, *p* < 0.01), which may be attributed to anaerobic conditions in waterlogged soils that can inhibit decomposition and nutrient mineralization despite high carbon stocks. Collectively, these findings indicate that while temperature (MAT), precipitation (MAP), and vegetation structure (LAI) are universally critical drivers, their relative importance and direction of influence diverge significantly across ecosystem types due to differing limiting factors and environmental adaptability.

### 3.3 Identify core variables and their influencing patterns

**Figure 4:**
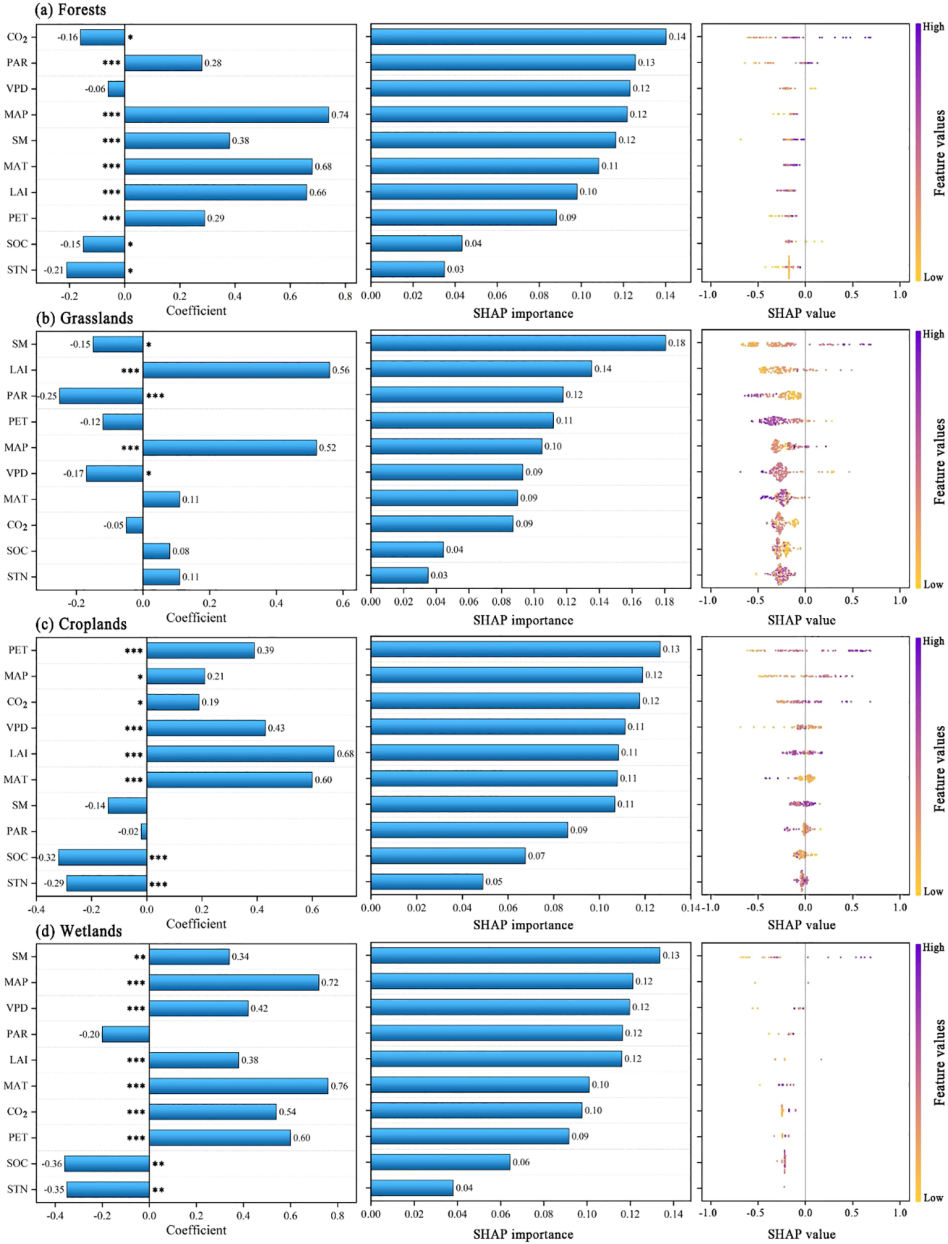
Relative importance and association patterns of AGPP-related variables across ecosystem types. Panels **(a–d)** show results for forests, grasslands, croplands, and wetlands, respectively. In each panel, the left plot presents the Pearson correlation coefficients between AGPP and the corresponding variables (*p* < 0.05; ** *p* < 0.01; *** *p* < 0.001). The middle plot shows the mean absolute SHAP values derived from the XGBoost model, indicating the relative importance of each variable within the statistical model. The right plot is the SHAP summary plot, illustrating how higher or lower values of each variable are associated with AGPP predictions. The color bar represents feature values.

By combining SHAP with Pearson correlation analysis, we found clear ecosystem-specific differences in the variables associated with AGPP (**Figure 4**). In forests, the SHAP analysis identified CO_2_, PAR, and VPD as the most important variables, with mean absolute SHAP values of approximately 0.14, 0.13, and 0.12, respectively. In contrast, Pearson correlation analysis showed that MAP, MAT, and LAI had the strongest positive linear relationships with AGPP. This contrast indicates that forest AGPP is associated with hydrothermal conditions, canopy structure, and atmospheric factors through nonlinear and context-dependent relationships that are not fully captured by simple bivariate correlations. In grasslands and wetlands, SHAP identified soil moisture (SM) as one of the most influential variables, with mean absolute SHAP values of approximately 0.18 and 0.13, respectively, highlighting the importance of water-related conditions in these ecosystems. However, the direction of the linear relationship between SM and AGPP differed among ecosystem types. SM was positively correlated with AGPP in forests (*r* = 0.38) and wetlands (*r* = 0.34), but weakly negatively correlated in grasslands (*r* = −0.15) and croplands (*r* = −0.14). This pattern suggests that, in open ecosystems, higher surface soil moisture may coincide with other limiting conditions, such as reduced radiation, lower temperature, or seasonal constraints, rather than exerting a uniformly positive influence on productivity. A notable discrepancy was observed between weak linear correlations and high SHAP importance for some variables, particularly CO_2_ in forests (Pearson’s *r* = −0.16 vs. SHAP rank = 1) and SM in grasslands (Pearson’s *r* = −0.15 vs. SHAP rank = 1). We do not interpret these discrepancies as evidence of direct ecological causation. Instead, they suggest that AGPP responses may involve nonlinear effects, interaction effects, and covariation among environmental gradients that are not resolved by linear correlation analysis alone. Overall, these results show that the dominant statistical associations of AGPP differ among ecosystem types, and that nonlinear modeling provides complementary insights beyond conventional correlation analysis.

**Figure 5:**
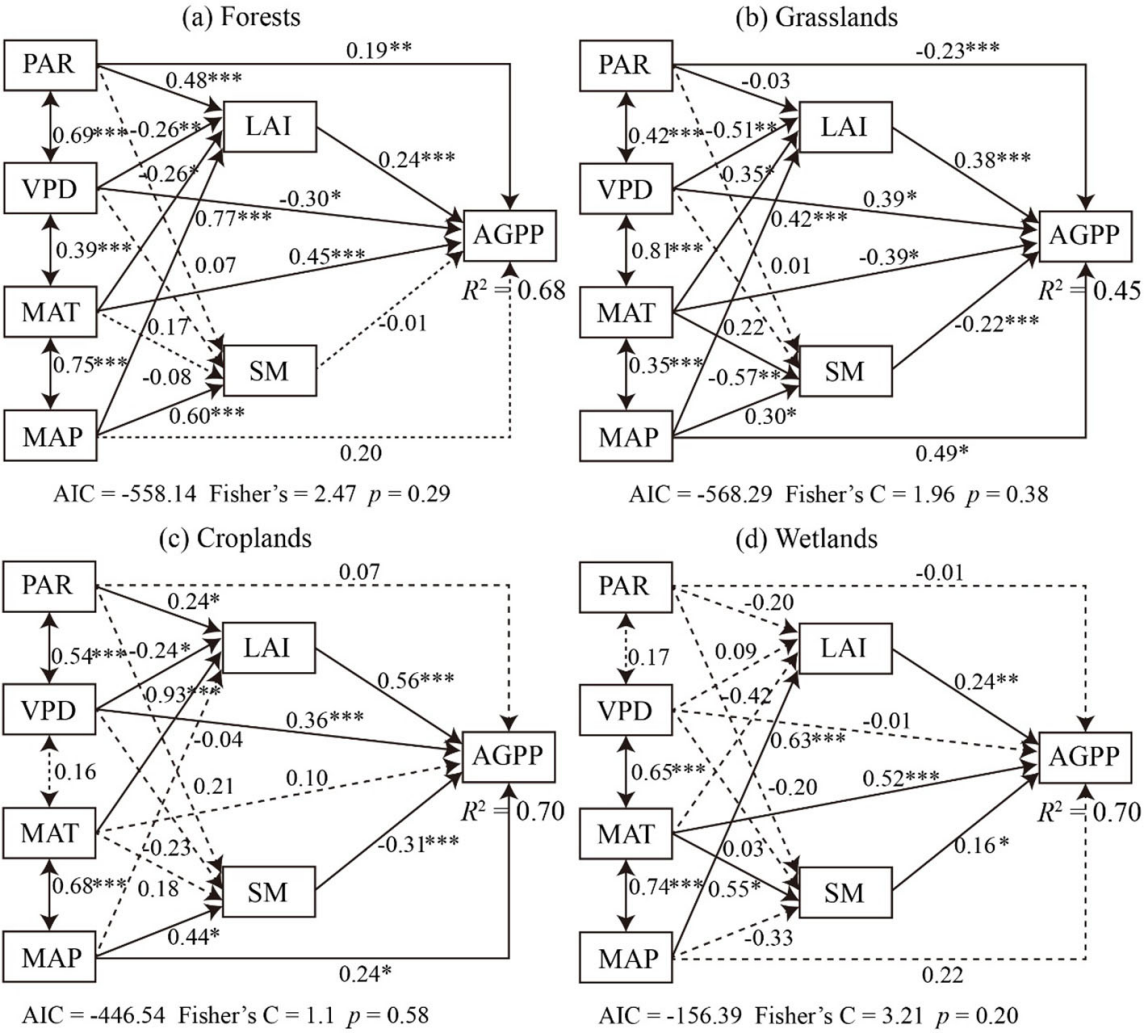
Structural equation models showing the pathway patterns linking environmental and biotic variables with AGPP across ecosystem types. Panels **(a–d)** represent forests, grasslands, croplands, and wetlands, respectively. Solid arrows indicate statistically significant paths, whereas dashed arrows indicate non-significant paths. Numbers adjacent to the arrows are standardized path coefficients (*p* < 0.05, ** *p* < 0.01, *** *p* < 0.001). Double-headed arrows indicate covariance between variables. R² values indicate the proportion of variance explained for each dependent variable. Model evaluation statistics (AIC, Fisher’s C, and p value) are shown below each panel.

**Figure 6:**
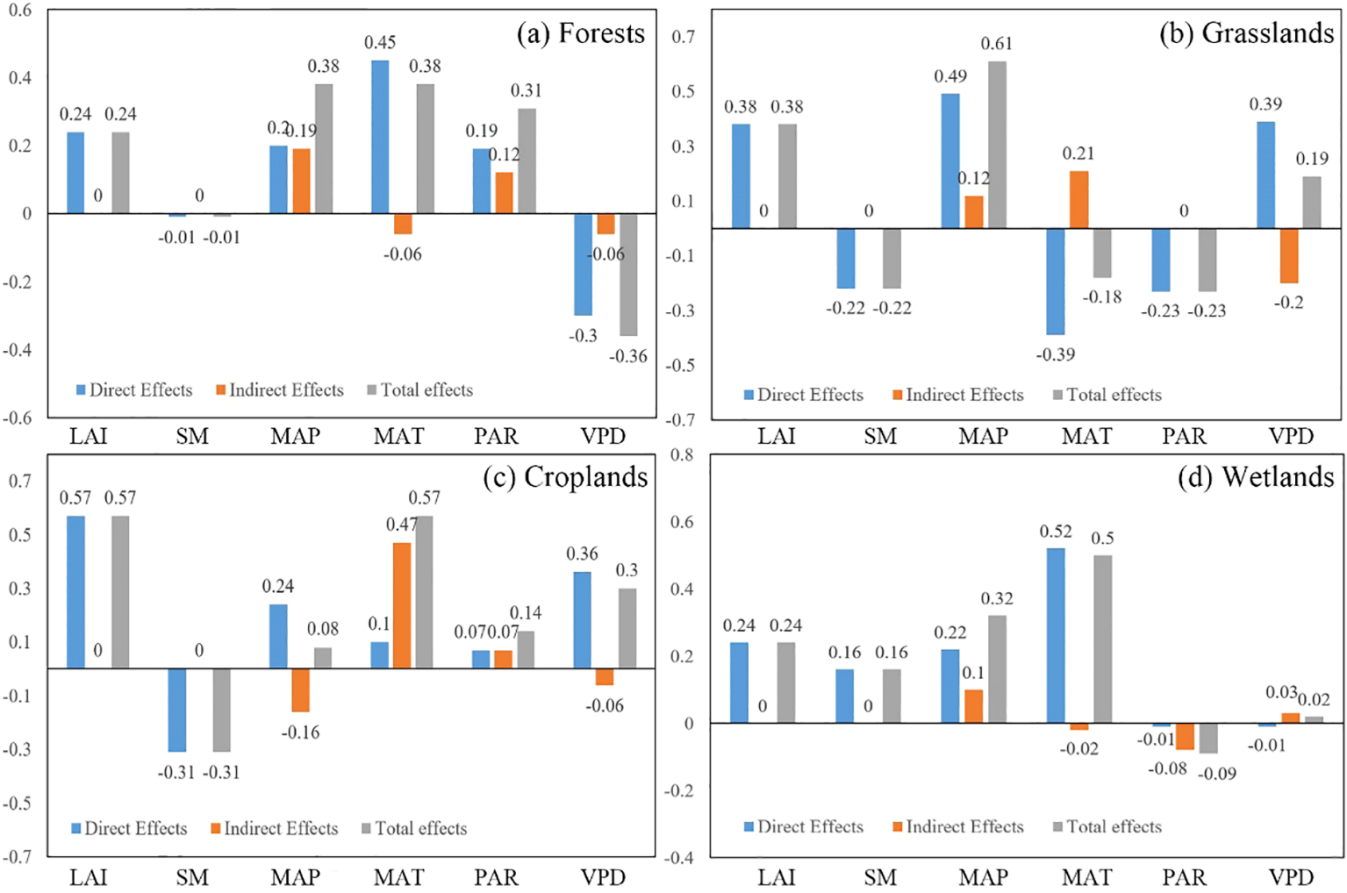
Standardized direct, indirect, and total effects derived from the structural equation models for different ecosystem types. Panels **(a–d)** show the SEM results for forests, grasslands, croplands, and wetlands, respectively. Blue, orange, and gray bars represent standardized direct, indirect, and total effects, respectively, illustrating the relative pathway contributions of AGPP-associated variables across ecosystem types.

Structural equation modeling (SEM) was further used to disentangle the direct and indirect pathways through which environmental and biotic variables were associated with AGPP across ecosystem types (**Figures 5**, **6**). Overall, the SEMs explained a substantial proportion of the variation in AGPP, with *R*² values ranging from 0.45 to 0.70. Although most key variables showed positive total associations with AGPP, the relative importance and pathway structure differed among ecosystems. In forests (**Figures 5a**, **6a**), AGPP was mainly associated with MAT, MAP, and VPD. MAT and MAP both showed positive total effects, whereas VPD showed a clear negative total effect (*β* = −0.36, *p* < 0.05). The effect of MAT was primarily direct, while the positive influence of MAP was partly mediated through increases in SM and LAI. These results suggest that forest productivity benefits from favorable thermal and moisture conditions, but that atmospheric dryness remains an important limiting factor. In grasslands (**Figures 5b**, **6b**), MAP and LAI were the variables most strongly associated with AGPP in the SEM framework. MAP had the strongest total effect, including both a direct positive effect and an indirect effect mediated through LAI. In contrast, PAR showed a significant negative effect on AGPP (*β* = −0.23, *p* < 0.001), indicating that higher radiation levels may coincide with stronger water limitation or evaporative stress in these relatively open ecosystems. In croplands (**Figures 5c**, **6c**), AGPP was most strongly associated with LAI and MAT, both of which showed positive total effects. The effect of LAI was predominantly direct, consistent with the close relationship between canopy development and crop productivity. By contrast, SM showed a negative total effect (*β* = −0.31, *p* < 0.001), suggesting that the role of soil moisture in croplands may be influenced by management-related factors and may not follow the same pattern observed in natural ecosystems. In wetlands (**Figures 5d**, **6d**), MAT and MAP showed the strongest total effects on AGPP. MAT mainly acted through a direct positive pathway, whereas the positive influence of MAP was partly mediated by SM. These results indicate that wetland AGPP is strongly associated with hydrothermal conditions and water-related pathways. Overall, MAT, MAP, and LAI emerged as recurrent variables associated with AGPP across China’s terrestrial ecosystems, although their pathway strengths and ecological roles differed among ecosystem types.

## 4 Discussion

### 4.1 Spatial patterns of AGPP and underlying environmental associations

From 2000 to 2020, annual gross primary productivity (AGPP) showed marked differences among terrestrial ecosystems in China. Forests exhibited the highest AGPP (1494.76 ± 39.16 g C m^−2^ yr^−1^), followed by wetlands (1181.21 ± 66.15 g C m^−2^ yr^−1^) and croplands (935.43 ± 26.18 g C m^−2^ yr^−1^), whereas grasslands showed the lowest values (442.18 ± 20.26 g C m^−2^ yr^−1^). This productivity hierarchy is broadly consistent with previous regional assessments. For example, Ma et al. (2025) reported that forestland NPP in Yunnan Province was substantially higher than that of grassland (936.17 g C m^−2^ yr^−1^) and cropland (942.49 g C m^−2^ yr^−1^), supporting the general pattern of higher productivity in forest ecosystems across heterogeneous landscapes.

The relatively high AGPP observed in forests was associated with favorable hydrothermal conditions and more developed canopy structure. Forest sites had the highest leaf area index (LAI: 2.41 ± 0.10 m² m^−2^), which is consistent with greater light interception capacity and photosynthetic potential. Previous studies have similarly shown that forest productivity in China is strongly associated with temperature, precipitation, radiation, and vegetation structure, with LAI functioning as an important intermediate variable linking environmental conditions to carbon uptake (Zhu et al., 2022, 2025). In this study, forests also showed relatively high MAT and MAP, further supporting the interpretation that both climate and vegetation structure are closely associated with their higher AGPP. Similar indirect effects of climatic conditions mediated through vegetation structure have also been reported in China’s drylands (Gong et al., 2024). Comparable linkages between macroclimate, vegetation structure, and productivity have also been documented in tropical secondary forests, further supporting the role of climate–vegetation interactions in shaping forest carbon uptake (Matsuo et al., 2025).

Wetlands also maintained relatively high AGPP, which was consistent with abundant water availability and comparatively stable thermal conditions (Wang et al., 2022). However, although wetlands contained high soil organic carbon (SOC) stocks, SOC was negatively correlated with AGPP. Rather than interpreting this pattern as a direct suppressive effect of SOC itself, it may indicate that waterlogged, high-carbon soils are associated with anaerobic conditions that constrain nutrient mineralization and root activity (Kayranli et al., 2010). This interpretation is consistent with previous studies reporting trade-offs between carbon storage and plant productivity in wetland and paddy-like systems (Xie et al., 2017). By contrast, grasslands showed the lowest AGPP, accompanied by the lowest LAI and relatively limited biomass accumulation. In these ecosystems, the negative correlation between soil moisture (SM) and AGPP suggests that higher surface soil moisture may not always coincide with favorable growing conditions. Instead, it may occur together with reduced radiation, lower temperature, or seasonal constraints in open ecosystems (Liu et al., 2020). Recent studies from grassland systems also support the view that the influence of soil moisture on productivity is context dependent and may vary with timing, soil depth, and climatic background (Nie et al., 2025; Liu Z. et al., 2025). This interpretation is also consistent with recent evidence showing that antecedent soil moisture conditions can exert stronger effects on ecosystem productivity than concurrent moisture status, highlighting the temporal complexity of water-related constraints on AGPP (Jiang et al., 2025).

Croplands showed intermediate AGPP levels and were characterized by stronger management influence than natural ecosystems. Although croplands had higher LAI than grasslands, the negative correlation between SOC and AGPP suggests that crop productivity may be partly decoupled from background soil properties under intensive management. Practices such as tillage, drainage, irrigation, and fertilization can modify soil conditions and sustain relatively high productivity even where soil carbon storage is comparatively low (Xu et al., 2024). In this sense, the observed SOC–AGPP relationship in croplands is more appropriately interpreted as a management-mediated pattern than as a simple soil–productivity relationship, which is also consistent with previous findings from intensively managed agroecosystems (Hu et al., 2021; Dold et al., 2017). Overall, the observed AGPP patterns across China’s terrestrial ecosystems were closely associated with ecosystem-specific combinations of climate, vegetation structure, soil conditions, and management effects. MAT and MAP were recurrent variables associated with higher AGPP, while LAI consistently reflected the role of vegetation structure in supporting carbon uptake (Zhu et al., 2022; Xia et al., 2015). At the same time, the ecological significance of soil-related variables differed among ecosystems, indicating that similar variables may operate through different pathways under different environmental and management contexts (Xu et al., 2024). In addition, previous studies have shown that synergistic effects of temperature and precipitation can enhance carbon uptake, whereas water limitation can offset these positive influences, reinforcing the importance of climate–water interactions in shaping productivity patterns (Wang Q. et al., 2015). These results are generally consistent with previous studies highlighting the importance of climate–vegetation interactions and ecosystem-specific constraints in shaping productivity patterns across China (Zhu et al., 2022; Gong et al., 2024; Ma et al., 2025).

### 4.2 Correlation patterns between AGPP and environmental factors and their ecological implications

Between 2000 and 2020, the relationship between annual gross primary productivity (AGPP) and environmental factors showed clear differences across China’s ecosystem types, revealing the distinct strategies vegetation uses to cope with environmental stress. In forest ecosystems, productivity was driven by a resource synergy of water and heat. AGPP showed strong positive correlations with MAP (*r* = 0.74) and MAT (*r* = 0.68). Coupled with a high LAI (*r* = 0.66), these conditions allow forests to maximize photosynthesis. Unlike other systems, forests also showed a positive link with SM (*r* = 0.38). This suggests that forests are the most resource-efficient systems: when water, heat, and soil moisture increase together, forest growth accelerates without the significant trade-offs seen in other ecosystems (Zhu et al., 2022). However, recent evidence indicates that forest GPP exhibits temperature thresholds beyond which productivity declines; for tropical forests, canopy temperatures above 28–29 °C result in declining rates of GPP increase with temperature, and significant GPP reductions occur at ~31 °C due to heat stress and stomatal closure (Pau et al., 2018). Similarly, temperate forests show optimal air temperatures for GPP around 20–27 °C, with reduced net carbon absorption at higher temperatures (Zeng et al., 2023), suggesting that the water-temperature synergy in forests has an upper thermal limit.

In contrast, grassland and wetland ecosystems faced critical environmental trade-offs. Grassland productivity was strictly limited by water (MAP, *r* = 0.52). A key finding was the negative link with sunlight (PAR, *r* = −0.25). This implies a “radiation paradox”: in semi-arid regions, high sunlight often comes with high evaporation and drought stress. To survive, plants must close their pores to save water, which stops growth even though there is plenty of light (Wang et al., 2022). This phenomenon is consistent with the “high-light stress” mechanism documented in drought-prone ecosystems, where excessive radiation combined with elevated vapor pressure deficit leads to photoinhibition and stomatal closure (De Boeck and Verbeeck, 2011). Recent research on the Tibetan Plateau further confirms that grassland water use efficiency is highly sensitive to soil moisture deficits, particularly under asymmetric daytime warming that exacerbates water stress (Du et al., 2025). Lü et al. (2025) demonstrated that warming and grazing in alpine meadows decrease net ecosystem productivity by 42.1%, primarily through increased aboveground respiration and reduced species richness, highlighting how combined stresses amplify the radiation-heat trade-off.

Wetland ecosystems relied heavily on a stable climate (MAT *r* = 0.76, MAP *r* = 0.72). While SM supported growth (*r* = 0.34), SOC had a strong negative effect (*r* = −0.36). This highlights a “nutrient lock” mechanism: wetlands store massive carbon stocks because the waterlogged soil lacks oxygen, which stops decomposition. However, this same lack of oxygen limits the release of nutrients needed for plant growth, meaning areas with the most carbon storage often have lower productivity (Xu et al., 2024). This trade-off is further complicated by vegetation-type effects on soil carbon fractions; plant communities with high water tables lead to anaerobic decomposition and reduced accumulation of labile organic carbon, while high-salinity environments inhibit microbial activity and slow decomposition (Li et al., 2025). Phosphorus availability in wetlands is particularly constrained under anaerobic conditions, as redox processes regulate P sorption and release, creating a “nutrient lock” that limits productivity despite high carbon stocks (Reddy et al., 2010). Additionally, rewetted peatlands show elevated CO_2_ and CH_4_ production when bioavailable organic matter is present, indicating that the carbon storage-productivity trade-off persists across different wetland types (Reuter et al., 2024).

Cropland ecosystems demonstrated how human activity can override natural rules. AGPP was driven by LAI (*r* = 0.68) and MAT (*r* = 0.60), but showed a significant negative link with SOC (*r* = −0.32). In natural systems, high soil carbon usually boosts growth. However, in croplands, heavy use of fertilizers and frequent tillage has decoupled productivity from soil health. This means high yields are currently maintained by human inputs rather than natural soil fertility (Wang F. et al., 2015). Long-term studies corroborate this decoupling effect: intensive tillage systems in Iowa showed SOC losses of 0.24–0.46 Mg C ha^−1^ yr^−1^, while no-till and strip-tillage systems gained 0.25–0.43 Mg C ha^−1^ yr^−1^, demonstrating that conventional management severs the productivity-soil carbon relationship (Lessmann et al., 2022). The conversion of grassland to cropland typically depletes SOC by 25–35%, and carbon-nutrient cycling becomes uncoupled due to the fixed stoichiometric ratios required for soil carbon sequestration (Olff et al., 2009). Halvorson et al. (2002) found that in the northern Great Plains, no-till annual cropping systems sequestered 233 kg C ha^−1^ yr^−1^ compared to a loss of 141 kg C ha^−1^ yr^−1^ with conventional tillage, highlighting how management intensity drives the negative SOC-AGPP relationship.

An additional point requiring careful interpretation is the weak negative Pearson correlation between atmospheric CO_2_ and AGPP observed in forests and grasslands. This pattern should not be interpreted as evidence that higher CO_2_ directly suppresses ecosystem productivity. Instead, it more likely reflects spatial covariation with other environmental gradients, such as drought stress, vapor pressure deficit, temperature, or site distribution effects. Because atmospheric CO_2_ in this study was analyzed together with multiple climatic and vegetation-related variables, its apparent bivariate relationship with AGPP may partly capture confounding spatial structure rather than a direct physiological response. This interpretation is also consistent with the discrepancy between the weak linear correlation and the relatively high SHAP importance of CO_2_, suggesting that its role is likely nonlinear, indirect, or dependent on interactions with other environmental conditions. These distinct mechanisms provide a roadmap for managing ecosystems under a changing climate. The strong reliance of forests and wetlands on synchronized rain and temperature makes them highly vulnerable to climate change; if warming occurs without more rain, these carbon sinks could collapse. This vulnerability is exacerbated by asymmetric daytime warming variation that have shifted from nighttime to daytime dominance since the 1980s, intensifying adverse impacts on productivity in dry and warm regions (Du et al., 2025). Therefore, conservation must focus on hydrological stability to buffer these shocks (Song et al., 2021). For grasslands, the results show that water is the absolute limit. Ecological restoration cannot just rely on planting grass but must focus on water conservation technologies to break the “radiation paradox” (Liu et al., 2020). This is particularly critical given that compound drought (combined vapor pressure deficit and soil water deficit) sensitivity is highest in grasslands and forests (Liu Y. et al., 2025). Finally, the negative soil-carbon link in croplands warns that future agriculture must shift toward conservation farming (like no-till) to reconnect high yields with healthy soil, ensuring food security against long-term degradation (Madani et al., 2020). Adoption of diversified crop rotations, cover crops, and organic amendments can help rebuild SOC stocks while maintaining productivity, as evidenced by systems that have achieved 0.2–0.6 t C ha^−1^ yr^−1^ sequestration rates through improved management (Lessmann et al., 2022).

### 4.3 Ecological significance and application value of key variables and their influence patterns

Between 2000 and 2020, the annual gross primary productivity (AGPP) of China’s terrestrial ecosystems was shaped by complex environmental conditions and ecosystem properties. By combining SHapley Additive exPlanations (SHAP) and structural equation modeling (SEM), we found that MAT, MAP, VPD, SM, LAI, and PAR were among the variables most strongly associated with AGPP. However, the effects of these factors differ greatly across ecosystems, indicating ecosystem-specific response patterns and regulatory pathways (Xu et al., 2024). A clear difference exists between forest and non-forest ecosystems. Forest ecosystems appeared to follow an atmospheric and energy-controlled mode. Our SEM results support this pattern: while MAT (*β =* 0.45) and MAP (*β =* 0.20) strongly enhanced AGPP, VPD has a significant negative effect (−0.30). This suggests that forests, having deep root systems, are less affected by short-term soil water shortages but are heavily limited by dry air. The strong positive correlations with MAP (*r* = 0.74) and MAT (*r* = 0.68) further suggest a hydrothermal synergy, meaning that higher temperatures only increase forest growth when there is enough rain (Xia et al., 2015). This matches recent global studies showing that higher VPD is a major threat from climate change, as drier air reduces photosynthesis and growth in many forest types (Novick et al., 2024). The inconsistency between Pearson correlation and SHAP importance for CO_2_ further indicates that simple linear correlations alone are insufficient to characterize its relationship with AGPP, especially when environmental feedbacks, nonlinear responses, and spatial covariation are involved.

In contrast, grassland and wetland ecosystems were more strongly associated with water- and soil-related constraints, showing complex physical trade-offs. Grasslands are very sensitive to a lack of rain (*r* = 0.52 for MAP) and face a unique radiation paradox. Although light is needed for photosynthesis, our data showed a significant negative effect for PAR (*β =* −0.23). This means that in dry areas, too much light causes severe water stress instead of growth, making high radiation a negative factor (Yang et al., 2023). Wetlands showed a potential nutrient-lock trade-off. Even though they store a lot of carbon, we found a significant negative link between soil organic carbon (SOC) and productivity (*r* = −0.36). Here, SOC should not be interpreted as a direct driver equivalent to climatic forcing, but rather as a soil property reflecting long-term anaerobic conditions, nutrient availability, and decomposition status. This suggests that the low-oxygen conditions needed to store carbon also stop nutrients from breaking down, which limits plant growth (Liu et al., 2020). Recent studies from restored wetlands in Northeast China support this: Zheng et al. (2025) showed that wetland restoration slows down how microbes use carbon. This happens because low-oxygen conditions change how key species interact, which helps store plant carbon in the soil but slows down decomposition. This balance between storing carbon and plant growth is kept by a simpler network of soil bacteria, leading to lower enzyme activity and slower SOC breakdown during restoration. Meanwhile, croplands reflected a human-managed production mode. The negative link between soil organic carbon (SOC) and productivity (*r* = −0.32) shows how heavy management, like fertilizers and irrigation, has separated crop yield from natural soil health. As in wetlands, SOC here is better interpreted as a soil property associated with long-term soil condition rather than as a direct productivity driver. This pattern implies that productivity in croplands is influenced not only by intrinsic soil status but also by substantial human inputs (Cao et al., 2022). This decoupling has also been documented in global studies. Lessmann et al. (2022) found that better cropland management could store between 0.28 and 0.43 Gt C per year globally, though this varies by region depending on current practices. Long-term studies in the northern Great Plains by Halvorson et al. (2002) provide direct evidence: farming without tilling and planting continuously increased carbon storage by 233 kg C per hectare each year, compared to a loss of 141 kg under normal tilling. However, recent studies suggest being careful: while no-till farming reduces soil mixing, it might just keep carbon levels stable rather than increasing them in some areas, and lower yields in wet climates show the need for local solutions (Weber et al., 2024).

Understanding these ecosystem-specific associations and pathways provides a clear scientific guide for managing ecosystems. Management plans must fit the specific needs of each system. For forests, because of the strong negative effect of VPD (−0.30), management should focus on structure optimization. This means changing tree density and choosing drought-resistant trees to reduce the stress from dry air (Geng et al., 2022). For grasslands, the negative impact of PAR (*β =* −0.23) makes water conservation technologies necessary. Instead of just planting more grass, work should focus on keeping moisture in the soil to reduce evaporation and overcome the radiation paradox. For croplands, the negative link with SOC (*r* = −0.32) shows an urgent need for soil health reconnection through conservation farming, like no-till methods, to fix soil damage (Halvorson et al., 2002). Finally, for wetlands, which are very sensitive to climate factors (*r* > 0.70 for MAT/MAP), hydrological stability is the most important goal. Keeping water levels steady prevents drying, which would break the nutrient lock (Zheng et al., 2025). Overall, these findings suggest that data-informed and ecosystem-specific management may improve carbon sequestration potential while supporting sustainable ecosystem functioning (Madani et al., 2020; Wang et al., 2024).

Despite these ecosystem-specific insights, several limitations of the present study should be acknowledged. First, although the flux-site dataset covers multiple terrestrial ecosystems across China, site distribution is spatially uneven, and some regions are represented by fewer observations than others. In addition, only a subset of sites has relatively long observation records, which may influence the balance between spatial coverage and temporal consistency. Second, although SHAP provides a useful framework for quantifying variable importance in nonlinear models, SHAP-based importance reflects model-based associations rather than definitive ecological causality. Although SHAP can be used to explore interaction effects and potential response thresholds, the present study focused on identifying major variables associated with AGPP and comparing their relative importance and pathway structure across ecosystems. A more systematic investigation of higher-order interactions and threshold behavior warrants dedicated analysis in future work. Third, while the present analysis is based on multi-year site observations, the inference is primarily oriented toward ecosystem-specific spatial variation rather than national-scale temporal variation. Finally, although we added uncertainty-related analyses where feasible, uncertainty associated with sampling structure, model specification, and site representativeness should still be considered when interpreting the results.

## 5 Conclusions

This study investigated the spatial patterns and regulatory mechanisms of Annual Gross Primary Productivity (AGPP) across China’s terrestrial ecosystems from 2000 to 2020. A clear productivity gradient was observed, with forests showing the highest AGPP (1494.76 ± 39.16 g C m^−2^ yr^−1^), followed by wetlands (1181.21 ± 66.15 g C m^−2^ yr^−1^) and croplands (935.43 ± 26.18 g C m^−2^ yr^−1^), whereas grasslands exhibited the lowest values (442.18 ± 20.26 g C m^−2^ yr^−1^). Although mean annual temperature (MAT), mean annual precipitation (MAP), and vegetation structure represented by leaf area index (LAI) were broadly associated with higher AGPP across ecosystems, the dominant limiting conditions differed markedly among ecosystem types. Forest AGPP was primarily constrained by atmospheric water demand, with vapor pressure deficit (VPD) showing a strong negative total effect (*β* = −0.36). Grassland AGPP was highly dependent on water availability, with MAP exerting a strong positive total effect (*β* = 0.61), while excessive photosynthetically active radiation (PAR) had a negative effect (*β* = −0.23), indicating a radiation paradox under water-limited conditions. In wetlands and croplands, AGPP was negatively associated with soil organic carbon (SOC; *r* ≈ −0.32 to −0.36), suggesting that SOC should be interpreted as a soil property linked to nutrient availability and long-term soil conditions rather than as a direct forcing factor. In wetlands, this pattern may reflect nutrient immobilization under oxygen-limited conditions, whereas in croplands it may indicate the partial decoupling of productivity from natural soil functioning under intensive management. In summary, these findings highlight strong ecosystem-specific differences in the variables and pathways associated with AGPP. Recognizing these distinct ecological constraints can improve understanding of terrestrial carbon cycling and provide a scientific basis for ecosystem-specific management strategies aimed at sustaining carbon sequestration under climate change.

## Data Availability

Publicly available datasets were analyzed in this study. This data can be found here: https://www.scidb.cn/detail?dataSetId=b496b208f51e44fcaf326e8b0f792c34&version=V3.
